# Targeted Metabolomics Uncovers NorCA’s Role as a Potent Immunomodulator in Acute Pancreatitis by Promoting Macrophage Reprogramming and Efferocytosis

**DOI:** 10.3390/ijms27104421

**Published:** 2026-05-15

**Authors:** Lingju Chu, Lei Zhang, Xinyi Liu, Qingtian Zhu, Xiaowu Dong, Chenchen Yuan, Weiwei Chen, Xingmeng Xu, Jiajia Pan, Guotao Lu, Weijuan Gong, Weixuan Yang, Yanbing Ding, Yaodong Wang

**Affiliations:** 1 The First Clinical College, Dalian Medical University, Dalian 116000, China; lingjuchu@gmail.com; 2 Medical College, Yangzhou University, Yangzhou 225000, China; aoe3721@163.com (L.Z.); yzuliuxy@163.com (X.L.); 15371259681@163.com (Q.Z.); dxw2333@163.com (X.D.); 092320@yzu.edu.cn (C.Y.); cww1984@126.com (W.C.); 13151513320@163.com (X.X.); 090517@yzu.edu.cn (J.P.); gtlu@yzu.edu.cn (G.L.); wjgong@yzu.edu.cn (W.G.); ywx7802@163.com (W.Y.); 3 Suzhou Key Laboratory of Integrated Traditional Chinese and Western Medicine, Kunshan 215300, China

**Keywords:** NorCA, macrophage reprogramming, efferocytosis, resolution of inflammation

## Abstract

Acute pancreatitis (AP) is a severe inflammatory disorder with limited therapeutic options. Novel bile acids have emerged as potent immunomodulators, but the function of norcholic acid (NorCA) previously remained unknown. In this study, we identified NorCA’s role as a novel immunomodulator that alleviates acute pancreatitis through peroxisome proliferator-activated receptor α (PPARα)-mediated macrophage reprogramming and efferocytosis. Targeted metabolomics was performed on serum from patients with AP and caerulein-induced AP mice. The functional role and mechanism of NorCA were investigated using flow cytometry, immunofluorescence, efferocytosis assays, and network pharmacology, both in vitro and in vivo. Our findings indicate that NorCA levels were significantly elevated in both patients and mice with AP, correlating with disease severity and complications. NorCA treatment markedly reduced serum amylase/lipase and pancreatic histopathological damage in AP mice. Mechanistically, NorCA promoted M1-to-M2 macrophage reprogramming and enhanced efferocytosis of apoptotic cells. These effects were dependent on PPARα activation, as demonstrated by siRNA silencing and pharmacological antagonism. These findings position NorCA as a promising therapeutic candidate and severity-associated metabolite in AP.

## 1. Introduction

Due to advances in mass spectrometry-based metabolomics and reverse metabolomics, a “bile acid renaissance” has occurred over the past decade, leading to the identification of nearly 200 novel bile acid species [1,2,3]. Functional characterization of these metabolites has revealed that bile acids signal through multiple receptors, including VDR and CAR, beyond the established FXR and TGR5, thereby exerting pleiotropic immunomodulatory effects [4,5,6].

However, the role of bile acids in acute pancreatitis (AP)—a severe inflammatory disorder of the exocrine pancreas with high morbidity—remains largely unexplored. Macrophages play a central role in AP pathogenesis, with M1 macrophages driving tissue injury and M2 macrophages promoting resolution and repair [7,8]. Promoting M1-to-M2 reprogramming and enhancing efferocytosis (clearance of apoptotic cells) are promising therapeutic strategies for AP [9,10].

NorCA is a previously uncharacterized bile acid distinguished by the absence of a terminal methylene group in its side chain, which prevents glycine or taurine conjugation and fundamentally alters its receptor interaction profile [11,12]. NorCA exhibits markedly lower affinity for FXR and impaired TGR5 engagement compared with CA [13], and its biological functions, particularly in acute inflammation, remain largely unknown.

In a recent targeted metabolomics study of 326 AP patients, we identified NorCA as one of the most significantly altered bile acids in AP sera [14], while in the present study, we investigated its functional role and extended our previous observations to a murine AP model. Our findings demonstrate that NorCA promotes M1-to-M2 macrophage reprogramming, enhances efferocytosis, and protects against pancreatic injury through activation of the PPARα signaling pathway. These results identify NorCA as a previously unrecognized immunomodulator with therapeutic potential for acute pancreatitis.

## 2. Results

### 2.1. Changes in the Metabolic Components of Serum Bile Acids in a Mouse Model and Clinical Sample

**Figure 1:**
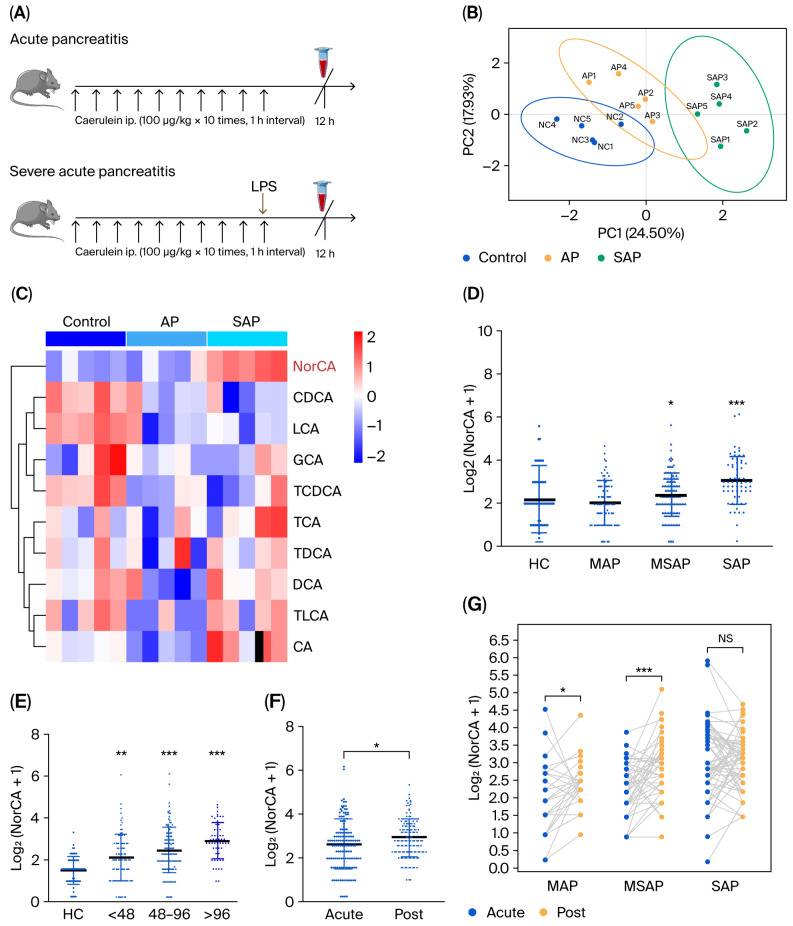
Changes in the metabolic components of serum bile acids in mouse model and clinical sample. (**A**) Schematic of targeted bile acid metabolomics in the mouse model. (**B**) PLS-DA plot of targeted bile acid metabolomics in the mouse model (*n* = 5 per group). (**C**) Heatmap of the mean normalized bile acid metabolite concentrations derived from targeted bile acid metabonomic profiling in the mouse model (*n* = 5 per group). (**D**) Scatterplots showing log2 transformation of the normalized concentrations of serum NorCA in patients with MAP (*n* = 99), MSAP (*n* = 144), and SAP (*n* = 83), and healthy controls (*n* = 60). (**E**) Scatterplots showing log2 transformation of the normalized concentrations of serum NorCA in acute-phase AP grouped according to time between onset and hospital admission (<48 h, *n* = 118; 48–96 h, *n* = 146; >96 h, *n* = 62) and healthy controls (HCs, *n* = 60). (**F**,**G**) Scatterplots displaying the changes in the normalized concentrations of serum NorCA in the acute phase (acute, *n* = 133) and recovery phase of AP (post, *n* = 133) in patients with MAP (*n* = 31), MSAP (*n* = 44), and SAP (*n* = 58). The data are presented as the means ± SD. *** *p* < 0.001, ** *p* < 0.01, and * *p* < 0.05. NS, not significant.

To investigate the alterations of bile acid metabolites in an AP mouse model, we performed a targeted metabolomic analysis of mouse serum, which included a total of 38 bile acid-related metabolites (Figure 1A). PLS-DA patterns were used to show the aggregation trends of the samples (Figure 1B). A total of 10 bile acids found in both humans and mice met the quality control criteria (Supplementary Table S1); their mean normalized concentrations are presented in the heatmap below (Figure 1C). We found that the concentration of NorCA in serum showed the most significant increase with increasing inflammation (Figure 1C), as reflected in volcano plots (Supplementary Figure S1A,B). We further evaluated NorCA levels in a sample of patients with AP. First, we reviewed clinical data from our recent study [12], including the general clinical and demographic data of healthy controls and patients with AP. Next, we examined the associations between serum NorCA and AP phase based on disease severity. The mean normalized concentrations of bile acid metabolites are presented in a heatmap (Supplementary Figure S1C), and fold changes in bile acid metabolite concentrations were visualized using Cleveland plots (Supplementary Figure S1D). Figure 1D demonstrates a significant correlation between AP severity and the changes in standardized serum concentrations of NorCA. Patients were then divided into three groups, reflecting time to hospital admission after the onset of acute AP, and the results show that the concentration of NorCA increased with time (Figure 1E). To assess the correlation between changes in serum bile acid metabolite levels and the course of AP, we compared NorCA levels in the acute phase and recovery phase. Interestingly, compared with the acute phase, NorCA levels did not decrease in the recovery phase but showed a further increase (Figure 1F,G).

Combining the quantitative results of bile acid metabolomics in the mouse model and clinical sample shown above, we found that NorCA levels were significantly correlated with the severity of AP. Notably, NorCA significantly increased during the acute phase of AP 4 and further increased during the recovery phase.

### 2.2. Correlation Between the Normalized Concentrations of Serum NorCA and the Clinical Features of Acute Pancreatitis Patients

**Figure 2:**
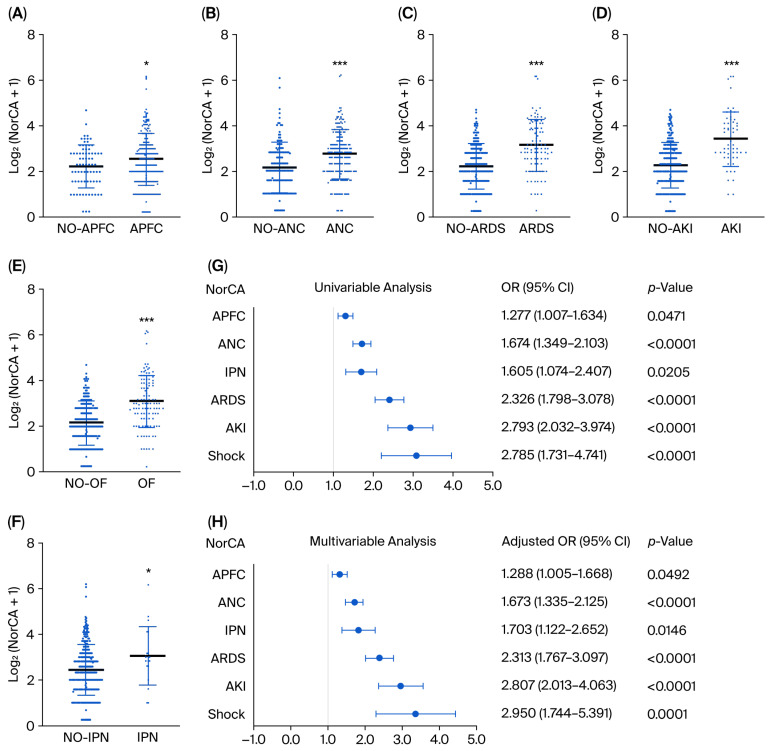
Correlation between the normalized concentrations of serum NorCA and the clinical features of acute pancreatitis patients. (**A**–**F**) Association analysis between the normalized concentrations of serum NorCA and APFC (APFC [*n* = 251] < NO-APFC [*n* = 75]), ANC (ANC [*n* = 186] < NO-ANC [*n* = 140]), ARDS (ARDS [*n* = 92], NO-ARDS [*n* = 234]), AKI (AKI [*n* = 53], NO-AKI [*n* = 273]), OF (OF [*n* = 108], NO-OF [*n* = 218]), and IPN (IPN [*n* = 19], NO-IPN [*n* = 307]). The data are presented as the means ± SD. *** *p* < 0.001 and * *p* < 0.05. (**G**,**H**) Forest plots of risk factors for APFC, ANC, IPN, ARDS, AKI, and shock in AP patients analyzed via univariate and multivariate logistic regression correcting for age, gender, body mass index, etiology, and disease parameters.

Next, we examined the associations between serum levels of NorCA and clinical features in the acute phase of AP, such as concurrent acute peripancreatic fluid accumulation (APFC), acute necrotic collection (ANC), infected pancreatic necrosis (IPN), and organ failure (OF). Figure 2A–F show that the levels of normalized serum NorCA were significantly higher in the groups with APFC, ANC, IPN, ARDS, AKI, and OF than in the control group. Univariate logistic regression analysis revealed that increased normalized NorCA levels were influencing factors for APFC, ANC, IPN, ARDS, AKI, and shock in patients with AP (Figure 2G). Furthermore, after correction for age, gender, BMI, etiology, and comorbid disease parameters, multivariate logistic regression analysis showed a similar result (Figure 2H). These data suggest that NorCA is significantly associated with AP severity and warrants further investigation as a candidate biomarker in prospective cohorts.

### 2.3. NorCA Protects Mice Against Experimental and Severe Acute Pancreatitis

**Figure 3:**
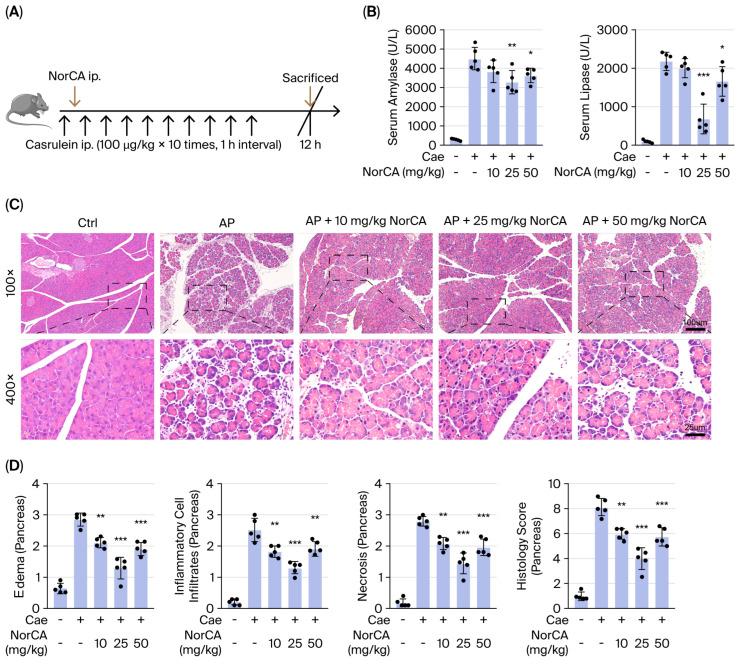
NorCA protects mice against experimental acute pancreatitis. (**A**) Schematic diagram of the caerulein-induced experimental AP model and NorCA intervention in mice. (**B**) Serum amylase and lipase levels at 12 h (*n* = 5 per group). (**C**) Hematoxylin and eosin (H&E) staining of pancreatic tissues from the indicated groups (*n* = 5 per group). Scale bar = 100 or 25 μm. (**D**) Histological scores (edema, inflammation, necrosis) of pancreatic tissues from AP mice (*n* = 5 per group). The data are presented as the means ± SD. *** *p* < 0.001, ** *p* < 0.01, and * *p* < 0.05 vs. the AP group.

We established a mouse model to further investigate the role of NorCA in AP (Figure 3A). The toxicity of NorCA (<50 mg/kg) to C57BL/6 mice was found to be negligible in a recent study [11]. After the addition of NorCA (10/25/50 mg/kg) in a dose gradient, the levels of serum amylase and lipase were markedly lower in the caerulein + NorCA group than in the AP group (Figure 3B). Furthermore, the degree of histological damage to the pancreatic tissue was significantly attenuated, with reductions in edema, infiltration of inflammatory cells, and necrosis of pancreatic acinar cells shown in the caerulein + NorCA group compared with the AP group (Figure 3C,D). The 25 mg/kg dose had the most significant protective effect against AP.

We also established a standard SAP mouse model via intraperitoneal injections of LPS in combination with caerulein (Supplementary Figure S4A). Similarly, the levels of serum amylase and lipase were markedly lower in the NorCA group than in the SAP group (Supplementary Figure S4B,C). The degree of histological damage to the pancreatic tissue was significantly attenuated in the NorCA group compared with the SAP group (Supplementary Figure S4D–H). Furthermore, atelectasis, pulmonary edema, inflammation, and congestion were also improved in the NorCA group, and the overall pathological damage to lung tissue was significantly reduced (Supplementary Figure S4I). The 25 and 50 mg/kg doses had the most significant protective effects against SAP.

In addition, treatment with NorCA one hour before caerulein injection also had a preventive effect on experimental AP (Supplementary Figure S2). Overall, our results show that all NorCA doses had a protective effect against (S)AP pathological injury.

### 2.4. NorCA Promotes Macrophage Reprogramming in Experimental and Severe Acute Pancreatitis

Since pancreatic acinar cells account for 95% of pancreatic tissues and their injury is considered a critical event in AP [15], we first verified whether NorCA had direct effects on acinar cells. Surprisingly, we found that NorCA had no effect on CCK-induced death in primary acinar cells (Supplementary Figure S3A). Neutrophils are also involved in AP through multiple effector mechanisms, such as the production of reactive oxygen species (ROS) [16]. We observed that NorCA also had no effect on in vitro neutrophil ROS production (Supplementary Figure S3B).

**Figure 4:**
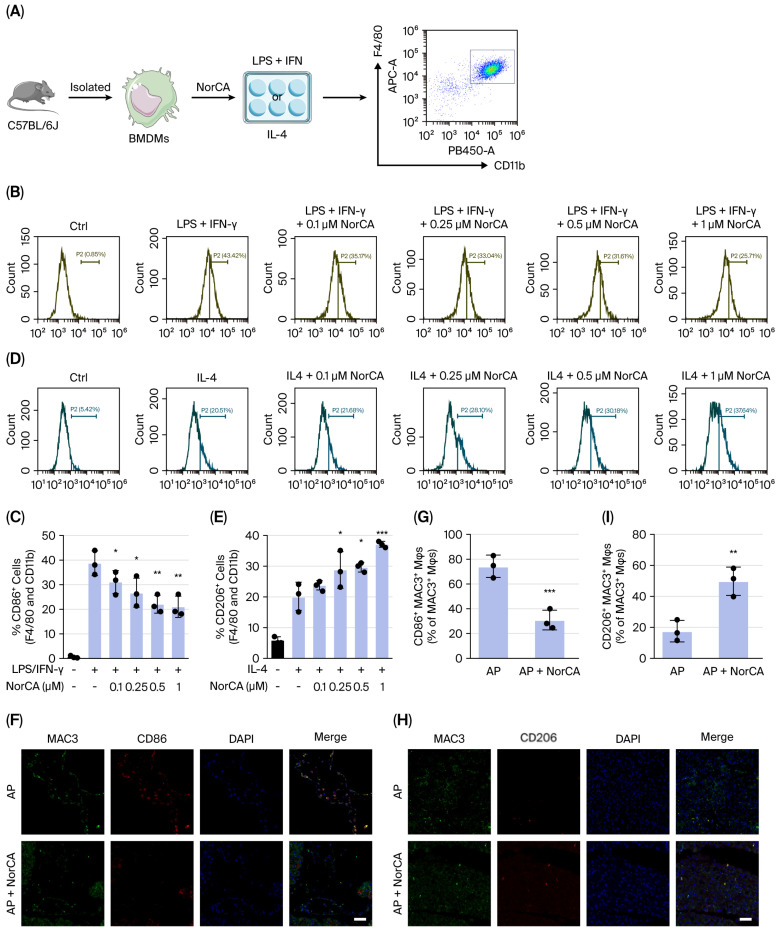
NorCA promotes macrophage reprogramming in experimental acute pancreatitis. (**A**) Schematic diagram of in vitro BMDM polarization induced by either IFN/LPS or IL-4 and NorCA intervention. (**B**,**C**) M1 markers in BMDMs were evaluated with flow cytometry (*n* = 3 per group). (**D**,**E**) M2 markers in BMDMs were evaluated with flow cytometry (*n* = 3 per group). (**F**,**G**) M1 markers were evaluated via immunofluorescence staining of tissue sections (*n* = 3 per group). (**H**,**I**) M2 markers were evaluated by immunofluorescence staining of tissue sections (*n* = 3 per group). The data are presented as the means ± SD. *** *p* < 0.001, ** *p* < 0.01, and * *p* < 0.05 vs. the LPS/IL-4 group. Scale bar = 25 μm.

Next, we verified whether NorCA had direct effects on macrophages. We performed in vitro polarization on bone marrow-derived macrophages (BMDMs) with either IFN/LPS to induce M1-like macrophages or IL-4 to induce M2-like cells (Figure 4A). BMDMs were treated with LPS (100 ng/mL) + IFN-γ (10 ng/mL) either alone or in combination with varying doses of NorCA (0.1, 0.25, 0.5, or 1 µM) for 24 h, which significantly altered cell phenotypes without cytotoxicity, and the results were compared with those of the no-treatment control group. Flow cytometry analysis revealed that, compared with the control group, LPS + IFN-γ significantly increased the expression of the M1 macrophage cell-surface marker CD86/CD80, while the addition of NorCA significantly decreased it (Figure 4B,C and Supplementary Figure S5A,C). Next, we treated BMDMs with IL-4 (20 ng/mL) either alone or in combination with NorCA (0.1, 0.25, 0.5, or 1 µM) for 24 h and compared the results with those of the no-treatment control group. Compared with the control group, IL-4 significantly increased the expression of the M2 macrophage cell-surface marker CD206/CD163, while the addition of NorCA further increased it (Figure 4D,E and Supplementary Figure S5B,D).

In addition, we investigated the effect of NorCA on macrophage polarization in vivo. Immunofluorescence staining was performed to explore macrophage infiltration in the pancreatic tissue sections. Intraperitoneal NorCA injection reduced the number of MAC3^+^CD86^+^/MAC3^+^CD80^+^ cells, representing the proinflammatory M1 phenotype (Figure 4F,G and Supplementary Figure S5E,F), and increased the number of MAC3^+^CD206^+^/MAC3^+^CD163^+^ cells, representing the pro-healing M2 phenotype (Figure 4H,I and Supplementary Figure S5G,H), resulting in an elevated ratio of M2/M1 macrophages.

### 2.5. NorCA Promotes Macrophage Efferocytosis in Experimental and Severe Acute Pancreatitis

**Figure 5:**
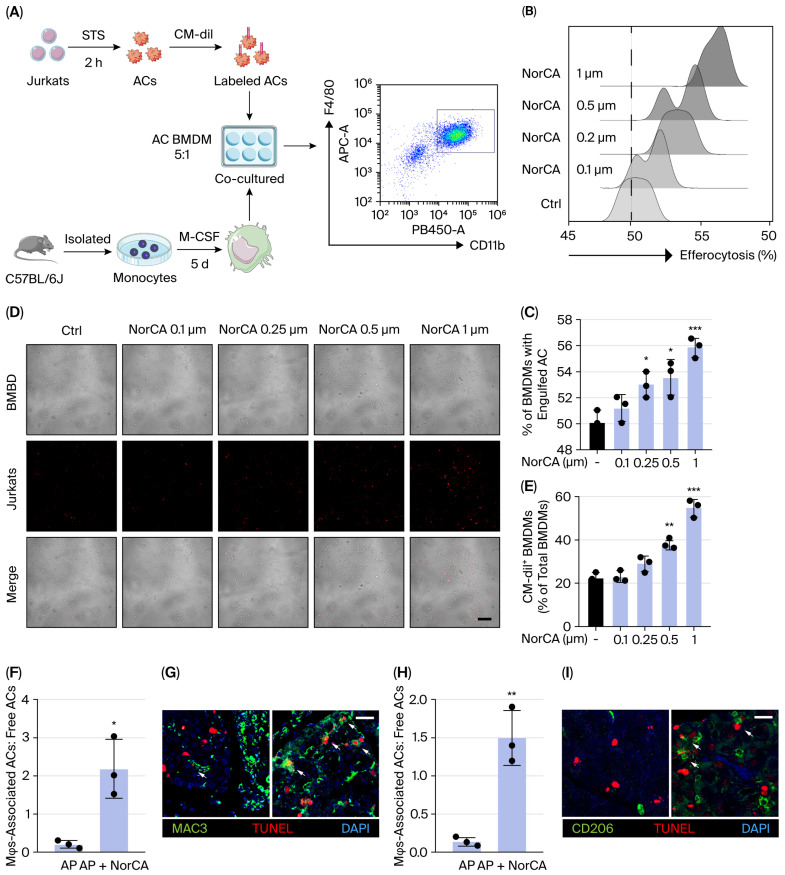
NorCA promotes macrophage efferocytosis in experimental acute pancreatitis. (**A**) Schematic diagram of the in vitro efferocytosis system. (**B**,**C**) In vitro efferocytosis was evaluated using flow cytometry and the quantity of CM-Dil^+^ ACs in CD11b^+^F4/80^+^ BMDMs (*n* = 3 per group). (**D**,**E**) In vitro efferocytosis was evaluated with laser confocal microscopy and the quantity of CM-Dil^+^ ACs in BMDMs (*n* = 3 per group). (**F**,**G**) The ratio of macrophage-associated ACs to free ACs was evaluated via immunofluorescence staining of pancreatic tissue sections (*n* = 3 per group). (**H**,**I**) The ratio of CD206^+^ macrophage-associated ACs to free ACs was evaluated via immunofluorescence staining of pancreatic tissue sections (*n* = 3 per group). The data are presented as the means ± SD. *** *p* < 0.001, ** *p* < 0.01, and * *p* < 0.05 vs. the control group. Scale bar = 25 μm.

Having established the role of NorCA in macrophage reprogramming both in vitro and in vivo, and given the importance of macrophages in efferocytosis, we further examined the impact of NorCA on apoptotic cell efferocytosis. CM-Dil, a red fluorescent probe, was used to label apoptotic Jurkat cells (ACs) to track the efferocytosis of macrophages (Figure 5A). Flow cytometry analysis revealed a significant increase in the efferocytic capacity of BMDMs after treatment with NorCA for 24 h (Figure 5B,C). These consistent results were also verified with fluorescence images obtained via laser confocal microscopy (Figure 5D,E).

To further evaluate efferocytosis in vivo, pancreatic tissue sections were assessed for the number of macrophage-associated ACs (TUNEL^+^), indicating macrophage phagocytic index. Consistent with the results of the in vitro phagocytosis assays, the ratio of macrophage-associated ACs to free ACs significantly increased in pancreatic tissue sections from NorCA-treated AP mice (Figure 5F,G), indicating that NorCA promoted efferocytosis. Further analysis revealed that CD206^+^ macrophage-associated TUNEL^+^ cells were present in NorCA-treated AP mouse tissue (Figure 5H,I). These data suggest that NorCA-mediated efferocytosis reprograms macrophages into an anti-inflammatory M2 phenotype and that M2 macrophages are involved in efferocytosis-induced pancreatic repair.

### 2.6. NorCA Upregulates Macrophage PPARα/γ Expression In Vitro and In Vivo

**Figure 6:**
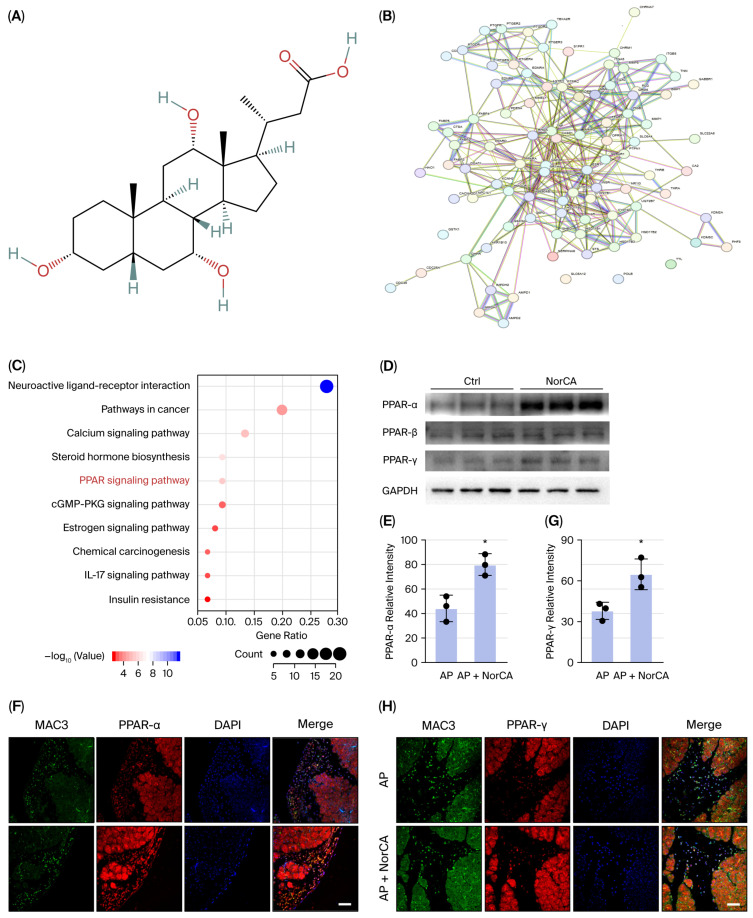
NorCA upregulates PPARα/γ expression of macrophages in vitro and in vivo. (**A**) Chemical structure of NorCA. (**B**) PPI network of all NorCA targets. (**C**) KEGG enrichment analysis of all NorCA targets. (**D**) PPARα/γ expression was detected via Western blotting. GAPDH was used as a loading control (*n* = 3 per group). (**E**–**H**) PPARα/γ expression was detected via immunofluorescence staining of pancreatic tissue sections (*n* = 3 per group). The data are presented as the means ± SD. * *p* < 0.05. Scale bar = 25 μm.

To further explore the mechanisms underlying the effects of NorCA in AP, we predicted potential targets using a structure-based network pharmacology analysis (Figure 6A and Supplementary Data S1). We constructed a PPI network to study the interactions between the targets, determined according to their prevalence in the network (Figure 6B). KEGG enrichment analysis was performed to investigate the signaling pathways relevant to the targets (Figure 6C). Of these, PPAR is of particular interest because it plays a critical role in efferocytosis and M2 anti-inflammatory macrophage skewing [9,17,18,19]. Our Western blot analysis revealed that NorCA significantly increased PPARα/γ protein levels in vitro (Figure 6D). Next, the role of NorCA in the AP mouse model was verified. The immunofluorescence data revealed that PPARα/γ levels were significantly increased in pancreatic tissue sections from NorCA-treated AP mice (Figure 6E–H). These results suggest that the PPAR signaling pathway may be involved in NorCA-mediated pancreatic repair.

### 2.7. NorCA Promotes Macrophage Reprogramming and Efferocytosis via Activation of PPARα Rather than PPARγ

**Figure 7:**
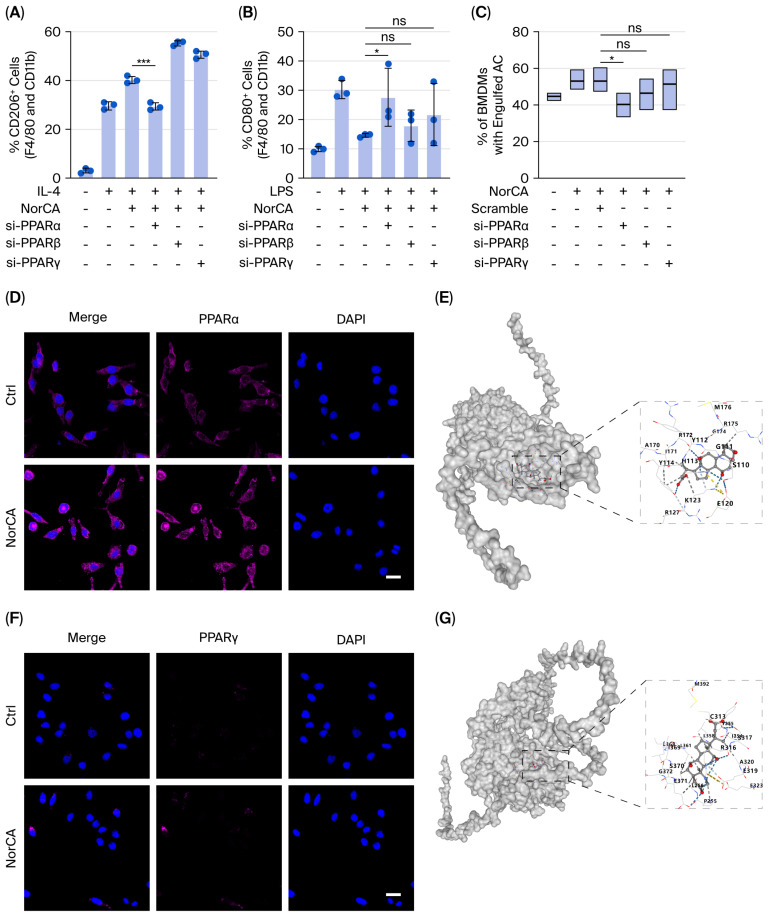
NorCA promotes macrophage reprogramming and efferocytosis via activation of PPARα rather than PPARγ. (**A**) M2 marker evaluation in BMDMs using flow cytometry (n = 3 per group). (**B**) M1 marker evaluation in BMDMs using flow cytometry (n = 3 per group). (**C**) In vitro efferocytosis was evaluated with flow cytometry. Efferocytosis was assessed based on the quantity of CM-Dil^+^ ACs in CD11b^+^F4/80^+^ BMDMs (*n* = 3 per group). (**D**) The expression and nuclear translocation of PPARα in BMDMs were detected via immunofluorescence staining (*n* = 3 per group). (**E**) Surface rendering of predicted NorCA binding mode on the active site of mouse PPARα. Interactions between NorCA and critical surrounding residues of PPARα are represented by sticks. (**F**) PPARγ expression in BMDMs was detected via immunofluorescence staining (*n* = 3 per group). (**G**) Surface rendering of predicted NorCA binding mode on the active site of mouse PPARγ. Interactions between NorCA and critical surrounding residues of PPARγ are represented by sticks. The data are presented as the means ± SD. *** *p* < 0.001, and * *p* < 0.05. ns, not significant. Scale bar = 25 μm.

We used siRNA to silence PPARα, PPARβ or PPARγ in BMDM cells, and then added NorCA, measuring the M1/M2 polarization and efferocytosis levels in macrophages. Supplementary Figure S6 shows the knockdown efficiency of PPARα/β/γ. Notably, we found that silencing PPARα, but not PPARβ or PPARγ, abolished the M1-to-M2 polarization transition (Figure 7A,B) and efferocytosis effect (Figure 7C) induced by NorCA. Immunofluorescence staining of BMDM cells showed that NorCA increases not only the total content of PPARα protein but also its nuclear localization (Figure 7D). These results suggest that NorCA promotes macrophage reprogramming and efferocytosis in a PPARα-dependent manner.

To investigate whether NorCA binds directly to PPARα, we used AlphaFold to dock NorCA into the structure of PPARα. The algorithm predicted that NorCA binds to the pocket of PPARα (Figure 7E), with a Vina score of -7.7 kcal/mol (Supplementary Data S2). Although the algorithm predicted that NorCA could also bind to PPARγ (Figure 7G and Supplementary Data S3, Vina score −8.2 kcal/mol), immunofluorescence showed that NorCA only slightly upregulated PPARγ protein levels but did not promote its nuclear translocation (Figure 7F). These data suggest that NorCA promotes macrophage reprogramming and efferocytosis via activation of the PPARα rather than PPARγ.

### 2.8. NorCA Promotes Reprogramming and Efferocytosis of Macrophages via PPARα Activation to Protect Mice Against (Severe) Acute Pancreatitis

**Figure 8:**
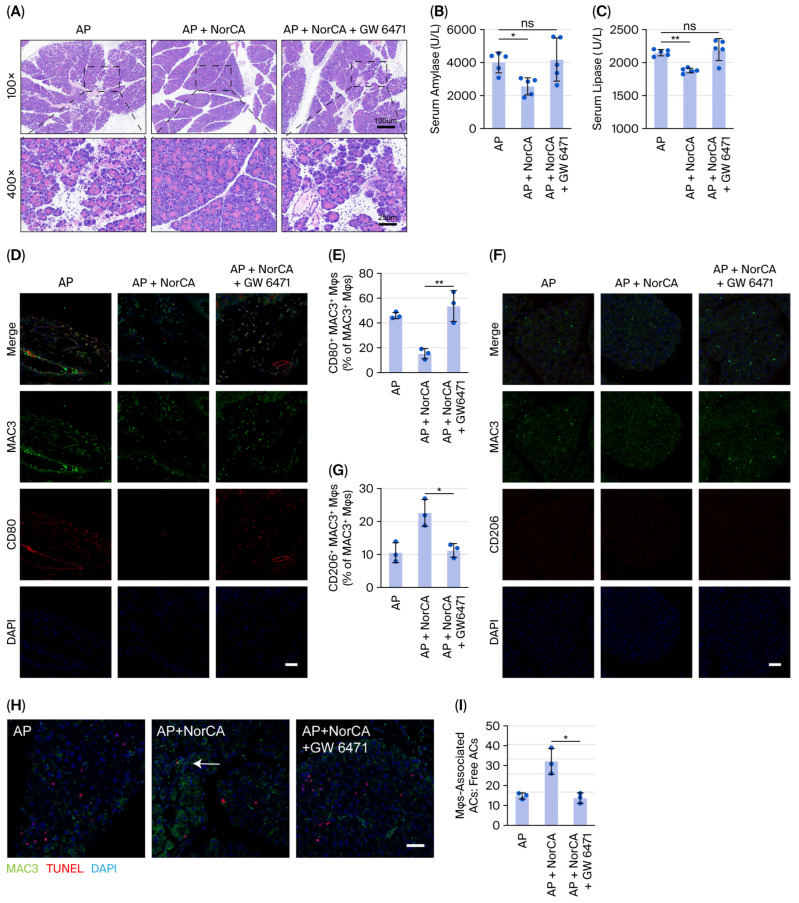
NorCA promotes reprogramming and efferocytosis of macrophages via PPARα activation to protect mice against acute pancreatitis. (**A**) H&E staining of pancreatic tissues from the indicated groups (*n* = 5 per group). (**B**,**C**) Serum amylase and lipase levels at 12 h (*n* = 5 per group). (**D**,**E**) M1 markers were evaluated via immunofluorescence staining of tissue sections (*n* = 3 per group). (**F**,**G**) M2 markers were evaluated via immunofluorescence staining of tissue sections (*n* = 3 per group). (**H**,**I**) The ratio of macrophage-associated ACs to free ACs was evaluated via immunofluorescence staining of pancreatic tissue sections (*n* = 3 per group). The data are presented as the means ± SD. ** *p* < 0.01 and * *p* < 0.05. ns, not significant. Scale bar = 100 or 25 μm.

We treated our (S)AP mouse model with the PPARα antagonist GW 6471 to further investigate the effects of NorCA in vivo. HE staining showed that GW 6471 removed the protective effect of NorCA on (S)AP mice at the histopathological level (Figure 8A and Supplementary Figure S7C,D). Moreover, the serum amylase and lipase indexes were consistent with the histopathological results (Figure 8B,C and Supplementary Figure S7A,B). Tissue immunofluorescence staining also revealed that GW 6471 stopped the NorCA-induced M1-to-M2 polarization transition (Figure 8D–G and Supplementary Figure S8A–D) and efferocytosis (Figure 8H,I and Supplementary Figure S8) in (S)AP mice. The above results demonstrate that NorCA promotes reprogramming and efferocytosis of macrophages via PPARα activation to protect mice against (severe) acute pancreatitis.

## 3. Discussion

Our findings in this work demonstrate that NorCA is a direct modulator of macrophage phenotypes and functions; importantly, it activated macrophage efferocytosis and, consequently, the inflammation resolution phenotype, showing therapeutic benefits including the amelioration of tissue damage and inflammation. Given its significant association with AP severity, further investigation of NorCA as a candidate biomarker in prospective cohorts is warranted.

Using bile acid metabolomics, we explored the role of NorCA and provided details concerning its effects on immunomodulation and pancreatic inflammation. Our data showed that NorCA treatment significantly accelerated macrophage M1-to-M2 reprogramming within pancreatic tissue after (S)AP and promoted efferocytosis to clear ACs via PPARα activation which, in turn, activated M2 macrophages to promote functional recovery. Furthermore, we suggest that NorCA promotes efferocytosis and macrophage reprogramming in (S)AP.

Our results demonstrate that NorCA levels are elevated in serum during AP (in both human and mouse models), with the increase being proportional to the severity of injury. Importantly, NorCA did not decrease in the AP recovery phase but showed a further increase. In addition, our animal experiments show that NorCA had a preventative as well as a therapeutic effect on AP pathological injury. These data suggest that although NorCA levels are elevated during AP to compensate, they may not be sufficient to alleviate inflammation, and therefore additional NorCA supplementation is needed to regulate inflammation and induce efferocytosis and alternate macrophage polarization.

When examining the protective effect of NorCA on AP mice, the 25 mg/kg dose showed the most significant effect compared with the 10 and 50 mg/kg doses, while in our SAP mouse model, doses of 25 mg/kg and 50mg/kg showed comparable protective effects. Several mechanisms may be involved in this phenomenon; as an endogenous compound of the body, it is unlikely that NorCA has potential toxicity. Therefore, 25 mg/kg may achieve the optimal effect–toxicity balance, while 10 mg/kg is insufficient and 50 mg/kg may lead to decreased efficacy due to the increased toxicity of solvents such as DMSO. This may be consistent with NorCA attenuating inflammation in a dose-dependent manner.

Primary bile acids undergo gut microbiota-mediated 7α-dehydroxylation in the colon, generating secondary species that comprise over 75% of circulating bile acids, dominating the systemic pool [4]. Functioning as endogenous ligands with spatiotemporal precision, bile acids exhibit dual-receptor activation capacity. They engage membrane receptors (e.g., TGR5/Gpbar1) for rapid signaling cascades and nuclear receptors (e.g., FXR/NR1H4) for transcriptional regulation. This bimodal receptor interaction underlies their pleiotropic immunomodulatory effects, regulating myeloid cell differentiation, lymphocyte activation, and inflammatory cytokine cascades through epigenetic and post-translational mechanisms. Acute inflammation resolution involves four coordinated mechanisms: biosynthesis of specialized pro-resolving mediators (SPMs), caspase-dependent neutrophil apoptosis, TAM receptor-mediated efferocytosis, and macrophage metabolic reprogramming [20]. IFN-γ/TLR4 activation activates M1 phenotypes with enhanced microbial clearance via ROS, while IL-4/IL-13 signaling activates M2 macrophages specialized in tissue repair and apoptotic cell clearance [21]. Phosphatidylserine recognition induces TGF-β/SMAD3 signaling, promoting anti-inflammatory reprogramming [22,23,24,25]. Efferocytosis modulation has demonstrated disease-modifying potential across chronic inflammatory and autoimmune conditions. Preclinical studies have validated its therapeutic utility in atherosclerosis, systemic lupus erythematosus, diabetic complications, and age-related inflammation [10,26]. Pharmacological innovation focusing on the efferocytosis potentiation and metabolic reprogramming of macrophages has therefore emerged as a strategic priority, given this dual capacity to attenuate pancreatic injury while promoting tissue remodeling. Pancreatic stellate cell deactivation and acinar regeneration [27] are critical transitions in the resolution of AP inflammation. Though it was previously unclear whether serum bile acids regulate macrophage efferocytosis during inflammation, our study has now filled this gap.

Peroxisome proliferator-activated receptors and ligand-inducible type II nuclear receptors transcriptionally regulate efferocytic machinery through direct binding to PPAR response elements in MERTK, CD36, and LXRA gene promoters [17,18,19]. Meanwhile, PPAR activation drives macrophage polarization toward anti-inflammatory M2 phenotypes via arginase-1/IL-10 upregulation [9,28]. Clinically, PPAR-targeted therapies like obeticholic acid (an FXR-PPARγ crosstalk agonist) demonstrate disease-modifying effects in cholestatic liver diseases [29]. Our network pharmacology analysis (Figure 6C) identified the IL-17 signaling and insulin resistance pathways as potential mediators of NorCA’s effects. The IL-17 pathway is particularly relevant to AP pathogenesis, as IL-17 promotes M1 macrophage polarization and neutrophil infiltration, while PPARα activation is known to negatively regulate IL-17-driven inflammatory responses [30,31]. The insulin resistance pathway links PPARα to metabolic reprogramming of macrophages—a process that underlies the transition from the proinflammatory M1 to the pro-resolving M2 phenotype [32]. Thus, although direct measurements of classic PPARα target genes (e.g., CD36, ABCA1) were not performed in the current study, the integrated bioinformatic and experimental evidence supports a model in which NorCA activates PPARα, which may in turn modulate IL-17 signaling and metabolic pathways to promote efferocytosis and M2 skewing. The main contribution of our study is our characterization of the previously unexplored norcholic acid, providing data on its immunomodulatory role in pancreatic inflammation. This finding has major implications and mechanisms for consideration in AP treatment.

## 4. Materials and Methods

### 4.1. Reagents

All reagents reported in this study are listed in the Supplementary Table S2 of the Supplementary Materials.

### 4.2. Human Subject Dataset Analysis

The human metabolomics datasets used in this study were retrieved from our prior investigation [12], with the bile acid profiling data publicly accessible through the Mendeley repository (https://data.mendeley.com/drafts/3dhymjr272), accessed on 21 October 2024.

### 4.3. Animals

Eight-week-old male C57BL/6J mice (20–25 g) were obtained from GemPharmatech (Suzhou, China) and acclimatized for 7 days to specific pathogen-free (SPF) conditions with controlled humidity (50 ± 5%), a 12/12 h light/dark cycle, and ad libitum access to standard chow and autoclaved water. All procedures were performed in full compliance with the ARRIVE guidelines. All animal experiments were conducted in accordance with the approval of the Laboratory Animal Ethics Committee of Yangzhou University (Approval No. 202404030).

### 4.4. Acute Pancreatitis Model Preparation and Drug Intervention

For acute pancreatitis (AP) induction, mice were randomly allocated into one of the following groups: vehicle control (5% DMSO in PBS, i.p.); caerulein-only AP treatment (100 μg/kg × 10 at hourly intervals, i.p.); and AP + NorCA treatment (10/25/50 mg/kg, i.p.). NorCA (CAS 60696-62-0) was administered 0.5 h post initial caerulein injection, followed by the PPARα antagonist GW 6471 (20 mg/kg, i.p.). To establish a severe AP (SAP) model, animals received caerulein (as above) followed by LPS (10 mg/kg, i.p.) with tissue collection at 12 h post-challenge. Mice were sacrificed under sodium pentobarbital anesthesia (50 mg/kg, i.p.) 12 h post-induction.

### 4.5. Sample Collection

Blood samples were obtained via retro-orbital venipuncture using heparinized capillaries and allowed to clot at room temperature for 30 min prior to centrifugation (2000× *g*, 15 min, 4 °C) to obtain serum. Serum amylase and lipase levels were quantified using the relevant kits according to manufacturer protocols. Pancreatic/pulmonary tissues were divided into two aliquots: one fixed in 4% paraformaldehyde for 24 h at 4 °C for histological processing, and the other snap-frozen in liquid nitrogen for molecular analyses.

### 4.6. Isolation and CCK Treatment of Primary Pancreatic Acinar Cells (PACs) and LDH Release Assay

PACs were isolated via sequential collagenase digestion (0.5 mg/mL in HBSS with 0.1% BSA) followed by density gradient centrifugation. Cells were maintained in HEPES-buffered DMEM (Sigma-Aldrich, St. Louis, MO, USA) with 0.01% soybean trypsin inhibitor. For CCK hyperstimulation, cells were pretreated with NorCA (0.1–25 μM) for 30 min prior to 10 μM CCK exposure (6 h). LDH release was quantified using LDH release kits following the manufacturer protocol.

### 4.7. Neutrophil Isolation and ROS Detection

Neutrophils were isolated using Histopaque discontinuous gradient (400× *g*, 30 min, brake off). Cell viability (>95%) was confirmed by trypan blue exclusion. For ROS detection, 5 × 10^5^ cells/well were preloaded with 10 μM DCFH-DA in HBSS for 30 min, followed by PMA (25 nM) stimulation ± NorCA in phenol red-free RPMI. Fluorescence intensity (Ex/Em 485/535 nm) was recorded every 5 min for 60 min using a SpectraMax i3x microplate reader (Molecular Devices, San Jose, CA, USA).

### 4.8. Isolation, Culture, and Characterization of Mouse BMDMs

BMDMs were generated by flushing femoral/tibial marrow with cold PBS through 25 G needles. Cells were cultured in RPMI 1640 supplemented with 10% FBS, 1% penicillin–streptomycin, and 20 ng/mL recombinant murine M-CSF. Medium was replaced on days 3 and 5. Flow cytometry confirmed >90% purity using CD11b-Pacific Blue™ (1:200, BioLegend, San Diego, CA, USA) and F4/80-APC (1:200, BioLegend, San Diego, CA, USA) with appropriate isotype controls.

### 4.9. Bile Acid Metabolomics

Targeted bile acid metabolomic profiling was conducted using a Waters ACQUITY UPLC system coupled with a XEVO TQ-S triple quadrupole mass spectrometer equipped with an electrospray ionization (ESI) source. Chromatographic separation was achieved on an ACQUITY BEH C18 column (2.1 × 100 mm, 1.7 μm) maintained at 40 °C. The binary mobile phase consisted of (A) 0.1% formic acid in LC-MS grade water and (B) 0.1% formic acid in acetonitrile, delivered at 0.3 mL/min using the following gradient program: 0–2 min 10% B, 2–8 min 10–95% B, 8–10 min 95% B. Mass spectrometric parameters were optimized using QuanOptimize (Waters, Milford, MA, USA) with the following settings: source temperature 150 °C, desolvation temperature 550 °C, capillary voltage 3.0 kV, and cone gas flow 150 L/h. Data acquisition employed multiple reaction monitoring (MRM) mode with compound-specific transitions and collision energies. The datasets reflecting the targeted bile acid metabolomics in our mouse model have been deposited in the Mendeley repository (https://data.mendeley.com/drafts/3dhymjr272, accessed on 14 October 2025) and are publicly available as of the date of publication.

### 4.10. Histological Examination of the Pancreas

Paraffin-embedded pancreatic sections (5 μm) were stained with hematoxylin and eosin using standard protocols. Histopathological scoring was performed by three board-certified veterinary pathologists blinded to experimental groups, employing the validated Schmidt scoring system with modifications: edema (0: absent, 1: interlobular, 2: intralobular, 3: bridging); necrosis (0: none, 1: <5%, 2: 5–20%, 3: >20% acinar involvement); inflammation (0: none, 1: perivascular, 2: <50% lobules, 3: >50% lobules). Total scores were calculated as the sum of individual parameters (range 0–9).

### 4.11. Efferocytosis Assessment

BMDMs were pretreated with NorCA (0.1–1 μM) for 24 h in complete RPMI 1640. Jurkat cells were labeled with 2 μM CM-Dil in serum-free medium (37 °C, 5 min) followed by quenching on ice. Apoptosis was induced with 2 μM staurosporine (STS) in 10% FBS-containing medium for 2 h (Annexin V+/PI+ >80% by flow cytometry). Efferocytosis was initiated by co-culturing CM-Dil+ Apoptotic Jurkat cells with BMDMs in a 5:1 ratio (37 °C, 5% CO_2_, 30 min). Non-phagocytosed ACs were removed with three PBS washes. Cells were fixed with 4% paraformaldehyde and imaged using Leica TCS SP8 confocal microscopy. Parallel samples were analyzed using flow cytometry (Beckman CytoFLEX S100EX, Beckman Coulter, Brea, CA, USA).

### 4.12. Immunofluorescence Staining of Pancreatic Tissue

Formalin-fixed paraffin-embedded sections underwent antigen retrieval in citrate buffer (10 mM, pH 6.0) at 95 °C for 20 min. After blocking with 3% normal goat serum in 0.3% Triton X-100/TBS, sections were incubated overnight at 4 °C with primary antibodies: Anti-CD86/80 (1:400), Anti-CD206/163 (1:400), and Anti-MAC3/CD107b (1:100). Secondary detection was performed with DyLight 488 (1:400) and Cy3-conjugated antibody (1:100). Slides were mounted with ProLong Diamond Antifade and imaged using sequential scanning mode to prevent spectral bleed-through.

### 4.13. In Situ Efferocytosis of Pancreatic Tissue

TUNEL staining was performed using a One Step TUNEL Apoptosis Assay Kit with the following modifications: sections were proteinase K-treated (20 μg/mL, 15 min) post-antigen retrieval. Fluorescent labeling employed Alexa Fluor 594-azide (1:500) in reaction buffer. Co-staining with CD206 (1:400) and MAC3 (1:100) was performed using the same protocol as the immunofluorescence staining of pancreatic tissue. Z-stack images (1 μm intervals) were acquired using 40× oil immersion objective. Efferocytic events were defined as CD206+ macrophages containing ≥3 TUNEL+ apoptotic bodies within 5 μm proximity, analyzed with Imaris v9.7 3D reconstruction software.

### 4.14. Flow Cytometry Analysis of Macrophage Polarization

BMDMs were polarized using the following cytokine regimens for 24 h. M1 polarization: 100 ng/mL LPS+ 10 ng/mL IFN-γ; M2 polarization: 20 ng/mL IL-4. Cells were pretreated with NorCA (0.1–1 μM) for 2 h prior to polarization induction. After 24 h of stimulation, cells were harvested and stained with fluorescent monoclonal antibodies (F4/80 and CD11b, CD86/80, CD206/163) for 30 min at 4 °C. Flow cytometry was performed on Beckman CytoFLEX and data acquisition maintained event rates < 1000 events/s.

### 4.15. siRNA Transfection

siRNA oligonucleotides were synthesized by GenePharma Co., Ltd. (Suzhou, China). siRNA (40 nM) was mixed with INTERFERin^@^ transfection reagent (Sartorius, Göttingen, Germany), and Opti-MEM (Gibco, Grand Island, NY, USA) was subsequently added and incubated for 10 min. The prepared mixture was added to plates lined with cells. The indicated target sequences are listed in the Supplementary Table S2.

### 4.16. Western Blot Analysis

Cellular proteins were extracted using RIPA lysis buffer supplemented with a protease/phosphatase inhibitor cocktail (1:100). Protein quantification was performed via BCA assay against bovine serum albumin standards. Denatured samples (20 μg/lane) were resolved on 10% gradient Tris–glycine gels at 110 V for 70 min followed by wet transfer (180 mA, 60 min) to 0.45 μm PVDF membranes (Merck Millipore, Darmstadt, Germany). Membranes were blocked with 5% non-fat dry milk in TBST (Tris-buffered saline + 0.1% Tween-20) for 1 h at RT, then incubated with primary antibodies overnight at 4 °C. After three TBST washes, membranes were probed with HRP-conjugated secondary antibodies (1:5000) for 1 h at RT. Chemiluminescent detection employed the SuperSignal™ West Femto Maximum Sensitivity Substrate (Thermo Fisher, Grand Island, NY, USA), with exposure times optimized using the ChemiDoc MP Imaging System (Bio-Rad, Hercules, CA, USA). Densitometric analysis was performed using Image J v1.7.0 software with GAPDH as the loading control.

### 4.17. Network Pharmacological Analysis

The molecular structure of NorCA (PubChem CID 158738) was curated using PubChem3D conformational analysis. Potential targets were predicted via SwissTargetPrediction with a probability threshold > 0.1. Target proteins were mapped to UniProtKB/Swiss-Prot entries using strict gene symbol matching. Protein–protein interaction networks were constructed using STRING DB (v11.5; confidence score ≥ 0.7). KEGG pathway enrichment was performed using Sangerbox tools (http://www.sangerbox.com).

### 4.18. Molecular Docking

The PDB format of the PPAR-α (AF-P23204-F1) and PPAR-γ (AF-P37238-F1) protein structure was downloaded from the UniProtKB database. The molecular structure of NorCA (PubChem CID 158738) was curated using PubChem3D conformational analysis. CB-Dock2 was used to identify the interaction between PPAR-α/γ and NorCA. The molecular docking was based on AutoDock Vina v1.2.7 and the potential binding sites of the query ligand was ranked according to the Vina score (kcal/mol). CB-Dock2 (RRID:SCR_026134) is an improved version of the protein–ligand blind docking tool that inherits the curvature-based cavity detection procedure and the AutoDock Vina-based molecular docking procedure in the CB-Dock server.

### 4.19. Quantification and Statistical Analysis

Metabolomics data preprocessing: Features with >50% missingness were excluded (QC-RSD < 20%). The remaining missing data were filled using the lower limit of the corresponding detection range of 0.5*. Log2-transformed data were normalized by probabilistic quotient normalization. Partial least-squares discriminant analysis (PLS-DA) was performed via the online OmicShare tools. Advanced heatmap plots and volcano plots were generated via OmicStudio tools. Paired *t*-tests were performed via Sangerbox tools. Flow cytometry analysis was performed with FlowJo v11.1.1. The statistical data are expressed as the means ± SDs. Experimental design followed at least three biological replicates with three technical replicates each. Student’s *t*-test was used for two-group comparisons; one-way ANOVA was used for multiple-group comparisons. Data visualization used GraphPad Prism 8.0 with raw data points superimposed on bar plots. Differences with *p* < 0.05 were considered statistically significant. * *p* < 0.05, ** *p* < 0.01, and *** *p* < 0.001.

## 5. Limitations of the Study

1. While our clinical analyses demonstrate significant associations between serum NorCA levels and AP severity, complications, and recovery-phase dynamics (Figure 1D–G and Figure 2A–H), these findings should be interpreted as hypothesis-generating rather than clinically definitive. Detailed biomarker validation includes determination of optimal diagnostic cut-off values, assessment of NorCA as an additive predictor beyond existing clinical scoring systems, time-dependent prognostic modeling, and validation in independent multicenter cohorts. Such analyses are essential to establish NorCA as a bona fide clinical biomarker and represent a clear priority for our ongoing and future work.
2. Although we demonstrated that NorCA alleviates inflammation via PPARα activation, it remains to be seen whether NorCA directly binds to PPARα, as molecular docking evidence alone is insufficient. If PPARα is a direct target of NorCA, the mechanism of how NorCA enters macrophages also remains to be elucidated. Is it passive diffusion or receptor-mediated endocytosis? Or are there transporters that assist entry into the cell? Therefore, we plan to further explore this mechanism in follow-up studies.
3. While our network pharmacology analysis identified the IL-17 and insulin resistance pathways as potential downstream mediators, the precise transcriptional effectors through which NorCA-activated PPARα promotes efferocytosis and M2 reprogramming were not directly validated in this study. Validation of specific targets remains a priority for our ongoing work.
4. Beyond (S)AP, we are also intrigued by the potential of NorCA in other inflammatory disease models. Future studies will explore its effects across diverse pathological conditions driven by inflammation, which could further elucidate its therapeutic and diagnostic potential.

## Data Availability

LC-MS/MS data and original Western blot images have been deposited in the Mendeley repository and are publicly available as of the date of publication. The DOI is listed in the Supplementary Table S2. All the data reported in this paper will be shared by the lead author upon request. Any additional information required to reanalyze the data reported in this paper is available from the lead author upon request.

## Supplementary Materials

The following supporting information can be downloaded at https://www.mdpi.com/article/10.3390/ijms27104421/s1.

## Abbreviations

NorCA	norcholic acid
Acs	apoptotic cells
PPAR	peroxisome proliferator-activated receptor
VDR	vitamin D receptor
CAR	constitutive androstane receptor
CCK	Cholecystokinin
LXRA	Liver X Receptor Alpha
CA	cholic acid
APFC	acute peripancreatic fluid accumulation
ANC	acute necrotic collection
IPN	infected pancreatic necrosis
OF	organ failure
ROS	reactive oxygen species
BMDMs	bone marrow-derived macrophages
PBC	primary biliary cholangitis
PSC	primary sclerosing cholangitis
SAP	severe acute pancreatitis
PACs	primary pancreatic acinar cells
M-CSF	macrophage colony-stimulating factor
STS	staurosporine
TUNEL	terminal deoxynucleotidyl transferase dUTP nick end labeling
MSAP	moderately severe acute pancreatitis
SPM	pro-resolving mediator
