# Supplementary material for: Targeted Metabolomics Uncovers NorCA’s Role as a Potent Immunomodulator in Acute Pancreatitis by Promoting Macrophage Reprogramming and Efferocytosis
Source: Int J Mol Sci. 2026 May 15;27(10):4421. doi: 10.3390/ijms27104421 (PMC13207563; doi:10.3390/ijms27104421)
Supplement: Supplementary file 1 [file ijms-27-04421-s001.zip › Supplementary Information 20260401.pdf]

Supplementary Information

Targeted Metabolomics Uncovers NorCA's Role as a Potent Immunomodulator in Acute Pancreatitis by Promoting Macrophage Reprogramming and Efferocytosis

Supplementary Figures and Figure Legends

Supplementary Fig. S1

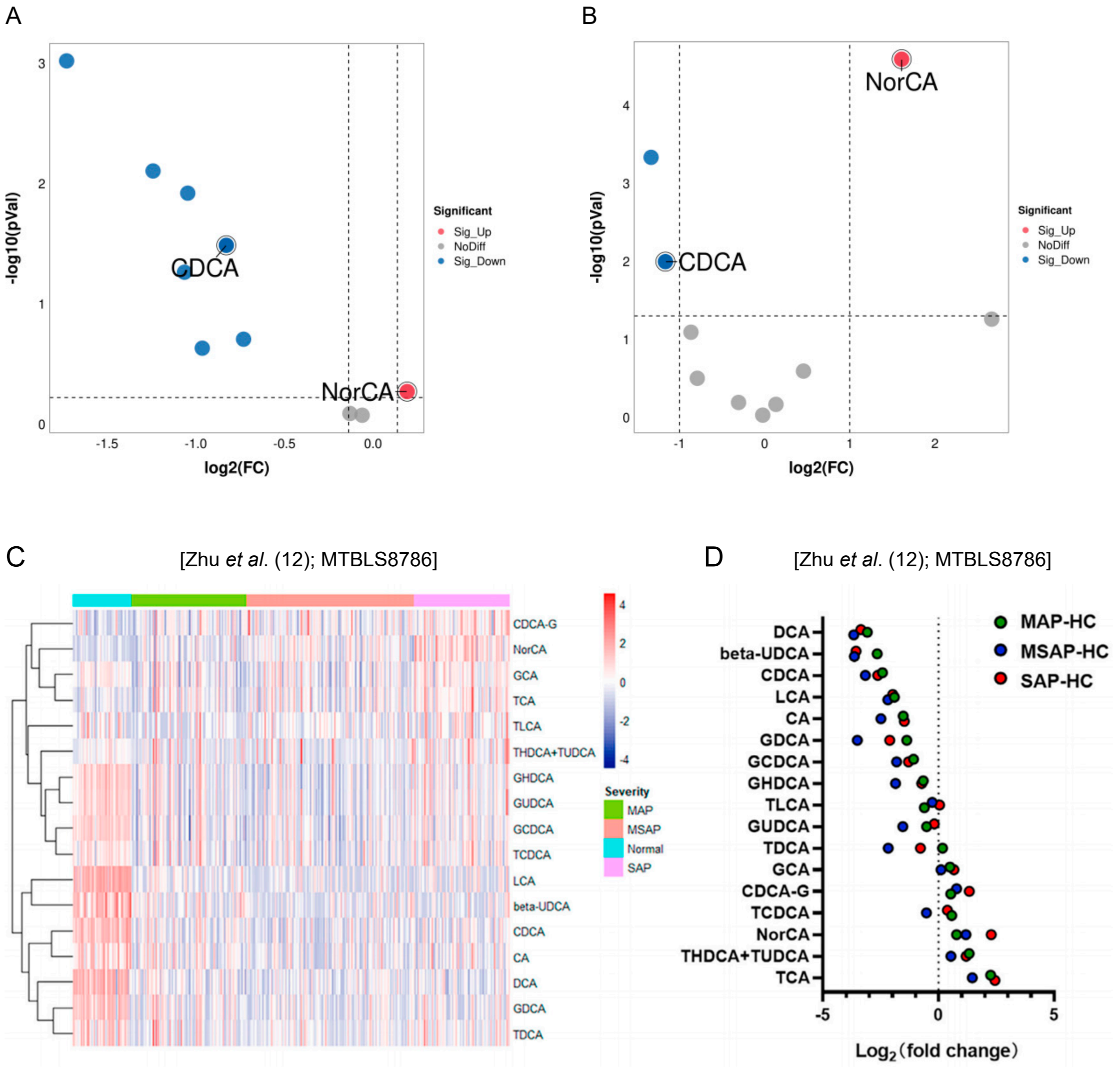

Supplementary Fig. S1 Volcano plots, Heatmap and Cleveland dot plot showed that the concentration of serum NorCA was significantly up-regulated in the inflammation group compared to the control group. Related to Figure 1

(A)Volcano plot representing the levels of 10 bile acids up- or down-regulated in AP mice model. (n = 5 per group)

(B)Volcano plot representing the levels of 10 bile acids up- or down-regulated in SAP mice model. (n = 5 per group)

(C)Heatmap of mean normalized bile acids metabolite concentrations derived from targeted bile acids metabonomic profiling dataset in the acute phase of AP patients (n = 326) and healthy controls (HCs, n = 60).

(D)Cleveland dot plot showing a ranked log2 transformation of fold changes of serum bile acids. Green, blue, and red dots represent the fold changes between patients with MAP (n = 99), MASP (n = 144), and SAP (n = 83), with respect to HCs (n = 60), respectively.

Data are presented as the mean ± SD. ***p<0.001, **p<0.01, and *p<0.05. NS, no significance.

Supplementary Fig. S2

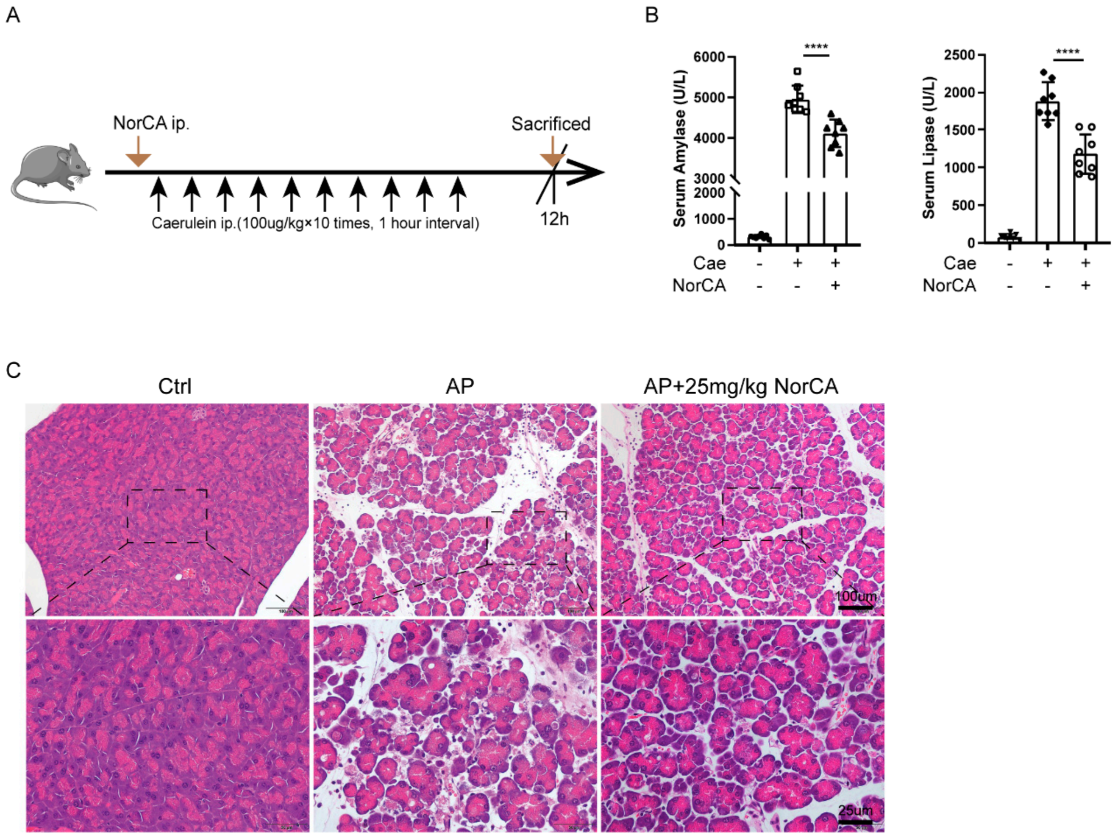

Supplementary Fig. S2 NorCA had a preventive effect on experimental AP. Related to Figure 3

(A)All groups except for the control group received intraperitoneal injections of caerulein (100 μg/kg, 1 h interval, 10 times) to induce the AP model. Drugs were administered 1 h before the first injection of caerulein in the NorCA+AP groups.

(B)Serum amylase and lipase levels at 12 hours (n = 8 per group).

(C)Hematoxylin and eosin (H&E) staining of pancreatic tissues from the indicated groups. Scale bar= 100 or 25 μm.

Data are presented as the mean ± SD. ****p<0.0001.

Supplementary Fig. S3

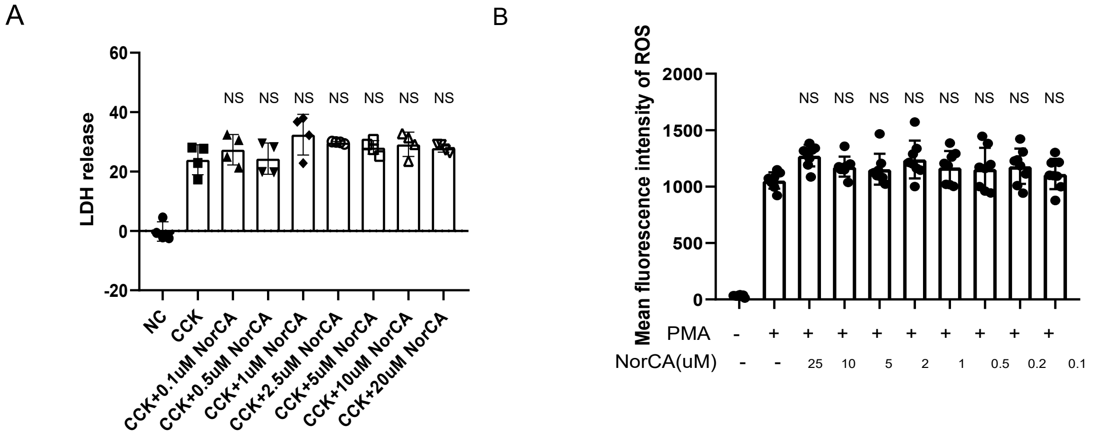

Supplementary Fig. S3 NorCA had no effect on CCK-induced cell death in primary acinar cells and NorCA also had no effect on neutrophil ROS production in vitro. Related to Figure 4

(A) Pancreatic primary acinar cells were treated with CCK, together with gradient doses of NorCA for 6 hours, respectively. The levels of LDH release were shown (n = 4 per group).

(B) Bone marrow neutrophils of C57BL/6J mice were treated with PMA, together with gradient doses of NorCA, respectively. The mean fluorescence intensity of ROS were shown (n = 7 per group).

Data are presented as the mean ± SD. NS, no significance vs CCK/PMA group.

Supplementary Fig. S4

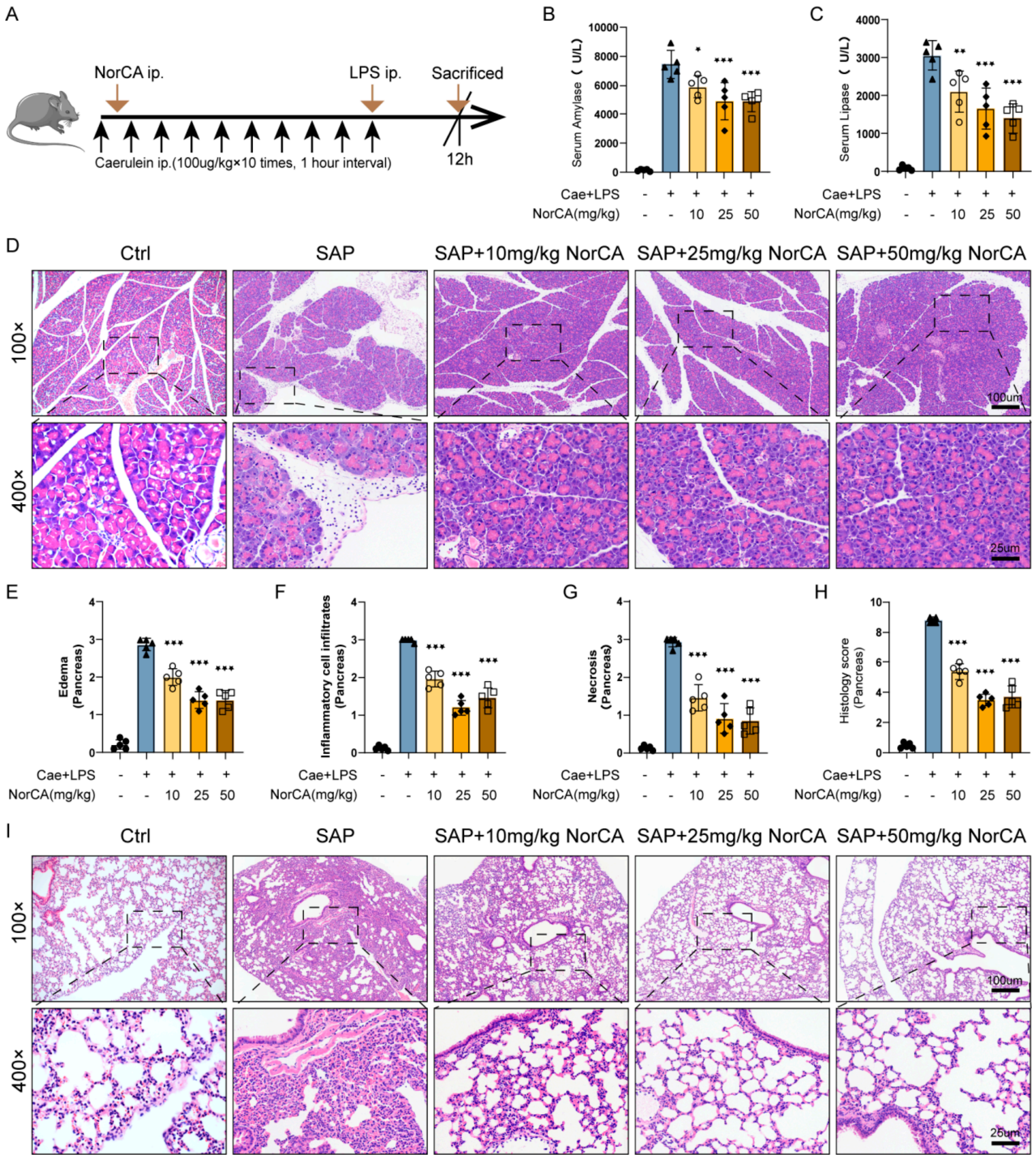

Supplementary Fig. S4 NorCA protects mice from experimental severe acute pancreatitis. Related to Figure 3

(A) Schematic diagram of the caerulein+LPS-induced experimental SAP model and NorCA intervention in mice.

(B and C) Serum amylase and lipase levels at 12 hours (n = 5 per group).

(D) H&E staining of pancreatic tissues from the indicated groups. (n = 5 per group).

(E-H) Histological scores (edema, inflammation, necrosis) of pancreatic tissues from SAP mice (n = 5 per group).

(I) H&E staining of lung tissues from the indicated groups. (n = 5 per group).

Data are presented as the mean ± SD. ***p<0.001, **p<0.01, and *p<0.05 vs SAP group. Scale bar= 100 or 25 μm.

Supplementary Fig. S5

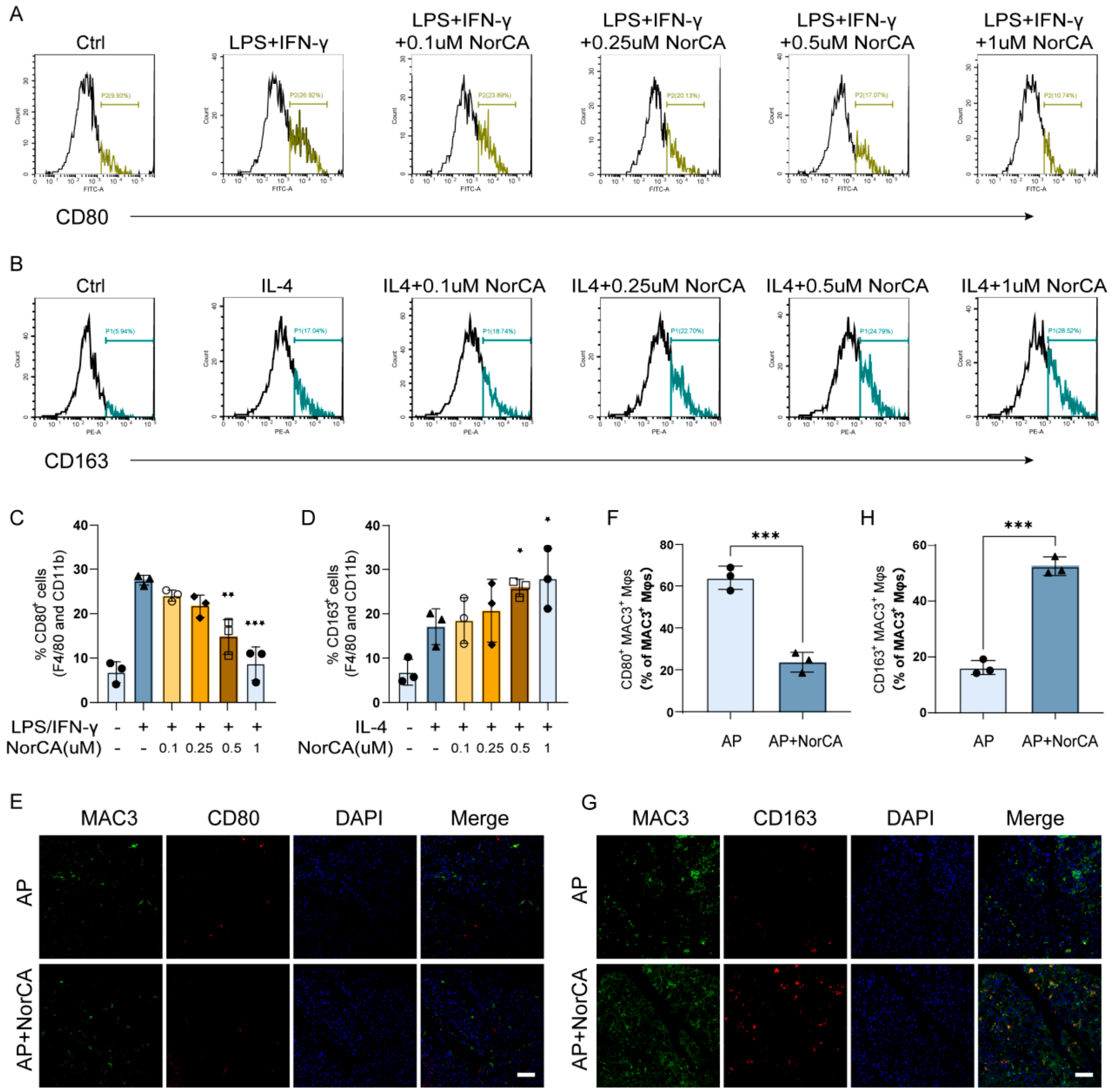

Supplementary Fig. S5 NorCA promotes macrophage reprogramming in experimental acute pancreatitis. Related to Figure 4

(A and C) M1 markers were evaluated by flow cytometry in BMDMs (n = 3 per group).

(B and D) M2 markers were evaluated by flow cytometry in BMDMs (n = 3 per group).

(E and F) M1 markers were evaluated via immunofluorescence staining of tissue sections (n = 3 per group).

(G and H) M2 markers were evaluated by immunofluorescence staining of tissue sections (n = 3 per group).

The data are presented as the means ± SD. ***p<0.001, **p<0.01, and *p<0.05 vs the LPS/IL-4 group. Scale bar= 25 μm.

Supplementary Fig. S6

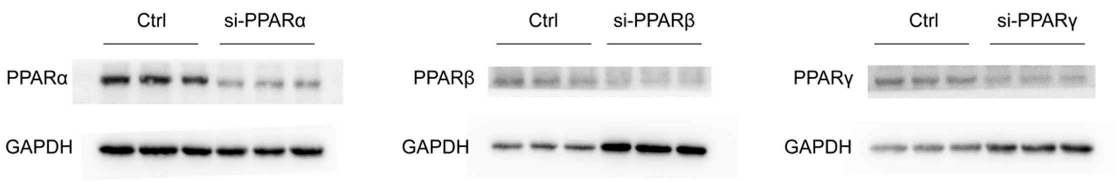

Supplementary Fig. S6 The knockdown efficiency of PPARα/β/γ in BMDM cells. Related to Figure 7

Representative images of western blotting for knockdown efficiency of PPAR-α/β/γ in BMDM cells (n = 3 per group).

Supplementary Fig. S7

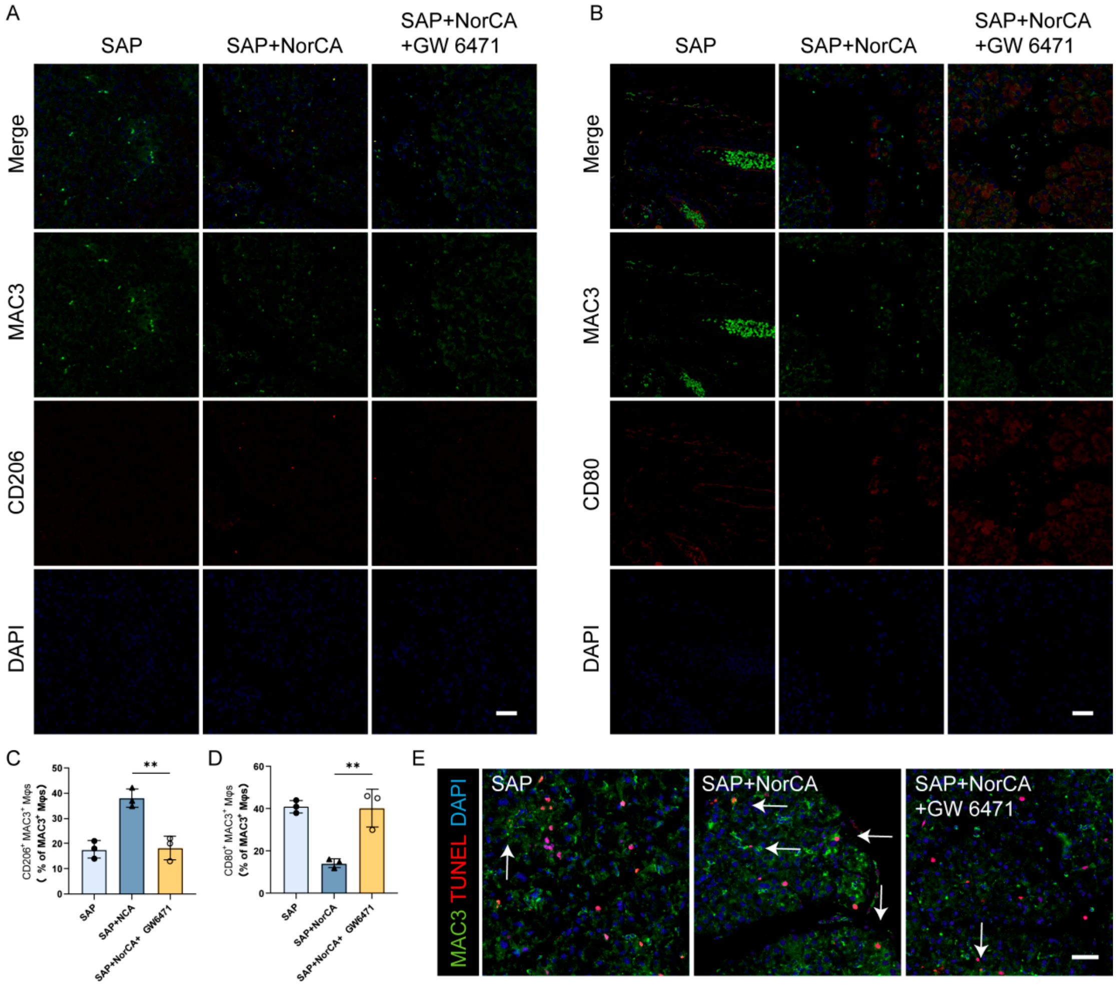

Supplementary Fig. S7 NorCA promotes reprogramming and efferocytosis of macrophages via PPARα activation. Related to Figure 4; Figure 5 and Figure 8

(A and C) M2 markers were evaluated via immunofluorescence staining of tissue sections (n = 3 per group).

(B and D) M1 markers were evaluated via immunofluorescence staining of tissue sections (n = 3 per group).

(E) The ratio of macrophage-associated ACs to free ACs was evaluated via immunofluorescence staining of pancreatic tissue sections (n = 3 per group).

The data are presented as the means ± SD. $^{**}p<0.01$. Scale bar= 25 μm.

Supplementary Fig. S8

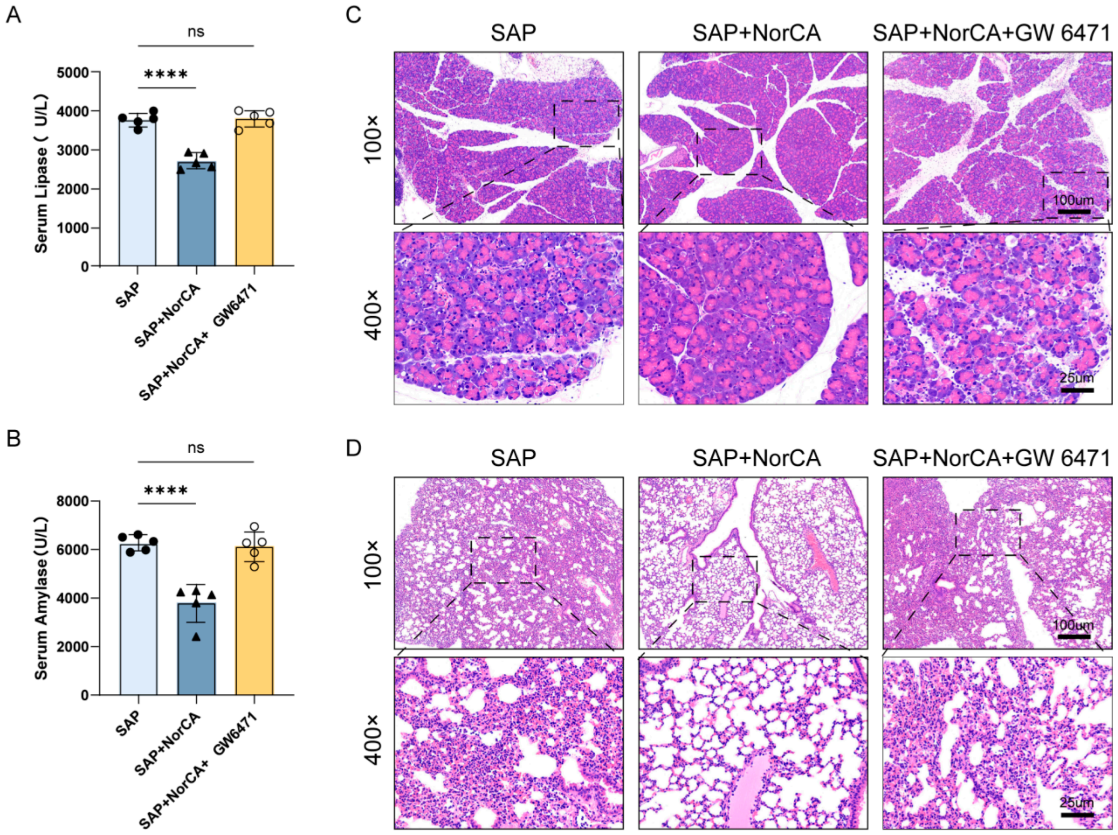

Supplementary Fig. S8 NorCA protects mice from severe acute pancreatitis via PPARα activation. Related to Figure 8

(A and B) Serum amylase and lipase levels at 12 hours (n = 5 per group).

(C) H&E staining of pancreatic tissues from the indicated groups. (n = 5 per group).

(D) H&E staining of lung tissues from the indicated groups. (n = 5 per group).

The data are presented as the means ± SD. ****p<0.0001. Scale bar= 100 or 25 μm.

Supplementary Table S1

No.	Abbreviation	Name	CAS
1	TCA	Taurocholic acid Sodium Salt	145-42-6
2	TCDCA	Taurochenodeoxycholic acid	6009-98-9
3	CA	Cholic acid	81-25-4
4	GCA	Sodium Glycocholate Hydrate	863-57-0
5	CDCA	Chenodeoxycholic acid	474-25-9
6	TDCA	Taurodeoxycholic acid Sodium Salt	1180-95-6
7	TLCA	Taurolithocholic acid Sodium Salt	6042-32-6
8	DCA	Deoxycholic acid	83-44-3
9	LCA	Lithocholic acid	434-13-9
10	NorCA	Norcholic acid	60692-62-0

Supplementary Table S2

REAGENT or RESOURCE	SOURCE	IDENTIFIER
Antibodies		
Anti-rabbit IgG, HRP-linked Antibody	Cell Signaling Technology	Cat#7074; RRID:AB_2099233
Anti-mouse IgG, HRP-linked Antibody	Cell Signaling Technology	Cat#7076; RRID:AB_330924
PPARγ (81B8) Rabbit mAb	Cell Signaling Technology	Cat#2443; RRID:AB_823598
PPARA Monoclonal antibody	Proteintech	Cat#66826-1-Ig; RRID:AB_2882169
PPARD Polyclonal antibody	Proteintech	Cat#28053-1-AP; RRID:AB_2918143
GAPDH (6C5) monoclonal antibody	Santa Cruz	Cat#sc-32233; RRID:AB_627679
Purified Rat Anti-Mouse CD107b (MAC3)	BD Pharmingen™	Cat#550292; RRID:AB_393587
Dylight 488, Goat Anti-Rat IgG	Abbkine	Cat#A23240; RRID:AB_3095855
CD206 Polyclonal antibody	Proteintech	Cat#18704-1-AP; RRID:AB_10597232
Cy3–conjugated Goat Anti-Rabbit IgG	Proteintech	Cat#SA00009-2; RRID:AB_2890957
APC anti-mouse F4/80 Antibody	BioLegend	Cat#123116; RRID:AB_893481
Pacific Blue™ anti-mouse/human CD11b	BioLegend	Cat#101224; RRID:AB_755986
PE Anti-Mouse CD86 (GL1)	Proteintech	Cat#PE-65068; RRID:AB_2883865
PE anti-mouse CD206 (MMR) Antibody	BioLegend	Cat#141706; RRID:AB_10895754
Alexa Fluor 488, Anti-Rat IgG	Abcam	Cat#ab150077; RRID:AB_2630356
FITC anti-mouse CD80 Antibody	BioLegend	Cat#104705; RRID:AB_313126
PE anti-mouse CD163 Antibody	BioLegend	Cat#155307; RRID:AB_2814061
Anti-CD163 antibody [RM1114]	Abcam	Cat#ab316218
CD80 monoclonal antibody	Santa Cruz	Cat#sc-376012; RRID:AB_10987889
Biological samples		
Blood samples from C57BL/6J mice	This paper	N/A
Pancreatic tissue samples from C57BL/6J mice	This paper	N/A
Lung tissue samples from C57BL/6J mice	This paper	N/A

Chemicals, peptides, and recombinant proteins		
Mouse M-CSF/CSF1 Recombinant Protein	TargetMOI	Cat#TMPY-00464
Mouse IL-4 Recombinant Protein	TargetMOI	Cat#TMPY-02558
Cholecystokinin (CCK)	MedChemExpress	Cat#HY-P0093
Phorbol-12-myristate-13-acetate (PMA)	MedChemExpress	Cat#HY-18739
Norcholic acid	MedChemExpress	Cat#HY-N9457
Taurocholic acid	MedChemExpress	Cat#HY-N0545R
Taurochenodeoxycholic acid	MedChemExpress	Cat#HY-N1429R
Cholic acid	MedChemExpress	Cat#HY-N0324R
Glycocholic acid	MedChemExpress	Cat#HY-N1423AR
Chenodeoxycholic Acid	MedChemExpress	Cat#HY-76847R
Taurodeoxycholic acid	MedChemExpress	Cat#HY-128853R
Taurolithocholic acid	MedChemExpress	Cat#HY-113308AR
Deoxycholic acid	MedChemExpress	Cat#HY-N0593R
Lithocholic acid	MedChemExpress	Cat#HY-B0172R
Caerulein	Sigma Aldrich	Cat#C9026
DMSO	Solarbio	Cat#D8370
Lipopolysaccharide (LPS)	MedChemExpress	Cat#HY-D1056
Staurosporine (STS)	YEASEN	Cat#50606ES03
Collagenase I	Sigma Aldrich	Cat#SCR103
Histopaque	Sigma Aldrich	Cat#11191/10771
DCFH-DA	Sigma Aldrich	Cat#D6883
ProLong Diamond Antifade	Thermo Fisher	Cat#P36962
GW 6471	TargetMOI	Cat#T8486
INTERFERin@ transfection reagent	Sartorius	Cat#101000028
Celltracker CM-DiI	YEASEN	Cat#40792ES50
Critical commercial assays		
LC-MS analysis	Wayen Biotechnologies, Inc	N/A
LDH Cytotoxicity Assay Kits	Beyotime	Cat#C0016
Mouse bone marrow neutrophil isolation kit	Tbdscience	Cat#TBD2013NM
Amylase Detection Kit	BioSino BioTechnology & Science	Cat#100000060
BCA Protein Assy Kit	Beyotime	Cat#P0012
One Step TUNEL Apoptosis Assay Kit	Beyotime	Cat#C1089
Lipase Detection kit	Nanjing Jiancheng	Cat#A054-1-1
Deposited data		
Original western blot data	Mendeley Data	chu, lingju (2025), "Original western blots 20251014", Mendeley Data, V1, doi: 10.17632/x42d3mvfxh.1

Targeted bile acid metabolomics datasets of mice	Mendeley Data	chu, lingju (2025), “Targeted bile acid metabolomics ”, Mendeley Data, V3, doi: 10.17632/3dhymjr272.3
Human metabolomics datasets	MetaboLights	https://www.ebi.ac.uk/metabolights/MTBLS8786
Experimental models: Cell lines		
Jurkat cells	ATCC	Cat#TIB-152; RRID: CVCL_0367
Experimental models: Organisms/strains		
C57BL/6J mice	GemPharmatech LLC.	Cat#N000295; RRID:IMSR_GPT:N000295
BMDMs from C57BL/6J mice	This paper	N/A
Oligonucleotides		
siRNA-Ppara-Mus: CCCUGUUUGUGGCUGCUAUTT	GenePharma	N/A
siRNA-Ppard-Mus: GGACUGAGUUCUCUAUCCUTT	GenePharma	N/A
siRNA-Pparg-mus: GGAGCCUAAGUUUGAGUUUTT	GenePharma	N/A
siRNA-Negative control: UUCUCCGAACGUGUCACGUTT	GenePharma	N/A
Software and algorithms		
FlowJo	BD	RRID: SCR_008520
ImageJ	NIH	RRID:SCR_003070
GraphPad Prism 8.0	GraphPad Software	RRID: SCR_002798
OmicShare	GENEDENOVO	http://www.omicshare.com/tools; RRID:SCR_025711
OmicStudio	lc-bio	https://www.omicstudio.cn
Sangerbox	SangerBox	http://www.sangerbox.com/tool
SwissTargetPrediction	The SIB Swiss Institute of Bioinformatics	http://www.swisstargetprediction.ch/; RRID:SCR_023756
UniProtKB	The UniProt consortium	https://www.uniprot.org/; RRID:SCR_004426
CB-Dock2	YANG CAO LAB	http://cadd.labshare.cn/cb-dock2/; RRID:SCR_026134
STRING DB	STRING CONSORTIUM	https://cn.string-db.org/; RRID:SCR_005223
